# Congenital Atrial Septal Defect Presenting With Tamponade Physiology and an Associated Viral Illness

**DOI:** 10.7759/cureus.41558

**Published:** 2023-07-08

**Authors:** Angel Juarez, Gabriela F Jhon, Rami Khouzam, Randall Goodroe, Russell Stahl, Mohamed Faris

**Affiliations:** 1 Internal Medicine, Grand Strand Medical Center, Myrtle Beach, USA; 2 Cardiology, Grand Strand Medical Center, Myrtle Beach, USA; 3 Cardiothoracic Surgery, Grand Strand Medical Center, Myrtle Beach, USA

**Keywords:** pericardiocentesis, beck's triad, right ventricular failure, atrial septal defect (asd), cardiac tamponade

## Abstract

Cardiac tamponade is a serious clinical syndrome that often presents with the classic triad of hypotension, jugular vein distention and diminished or muffled heart sounds on auscultation (Beck’s Triad). This phenomenon occurs due to fluid accumulation in the pericardial space which compresses the heart, reduces cardiac output and may cause cardiogenic shock. In this report, we present a case of a 22-year-old female with a congenital atrial septal defect (ASD) and right ventricular failure with tamponade physiology with an associated viral illness.

## Introduction

Cardiac tamponade is a life-threatening emergency that may present with an indolent or rapid onset. This condition occurs when enough fluid accumulates, at a certain rate in the pericardial sac to compress the heart and lead to a decrease in cardiac output and shock [1,2]. The diagnosis of cardiac tamponade is clinical, requiring prompt recognition and treatment to prevent cardiovascular collapse and cardiac arrest. The classic symptoms of cardiac tamponade were first described by the American cardiothoracic surgeon, Claude Beck, in 1935. These symptoms, known as Beck’s Triad, are hypotension, decreased or muffled heart sounds, and jugular venous distention. Although this symptomatology is highly suggestive of the presence of tamponade physiology, only a small number of cases will have all three elements [3,4]. There are various etiologies that can lead to a tamponade physiology, such as a hemorrhage from a penetrating wound, myocardial infarction, tuberculosis, myocarditis, autoimmune disease, uremia, or neoplasms. All of these can induce pericardial fluid buildup requiring swift medical or surgical management.

## Case presentation

We present a case of a 22-year-old woman from Chiapas, Mexico with a medical history of childhood asthma who relocated to the United States in Myrtle Beach, South Carolina 3 years prior to presentation. Her chief complaint was a 2-week history of worsening shortness of breath and nonproductive cough. The patient reported increasing dyspnea on exertion for the previous 6 months which she attributed to a resurgence in her childhood asthma, for which she did not think to seek treatment. The patient presented however to an urgent care center eight days prior to admission in view of worsening of her symptoms. Chest X-ray done at that time was significant for enlarged cardiac silhouette and potential findings of viral pneumonia. The patient tested positive for influenza A during the urgent care visit. She was discharged with cough medication and oseltamivir, which she did not take because the medications made her feel nauseous.

**Figure 1:**
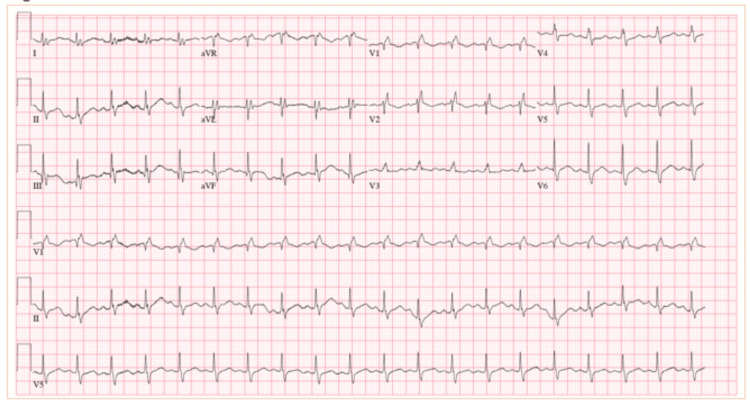
Initial EKG EKG shows sinus tachycardia with nonspecific ST-T-wave changes and significantly low amplitude and right bundle branch block.

**Figure 2:**
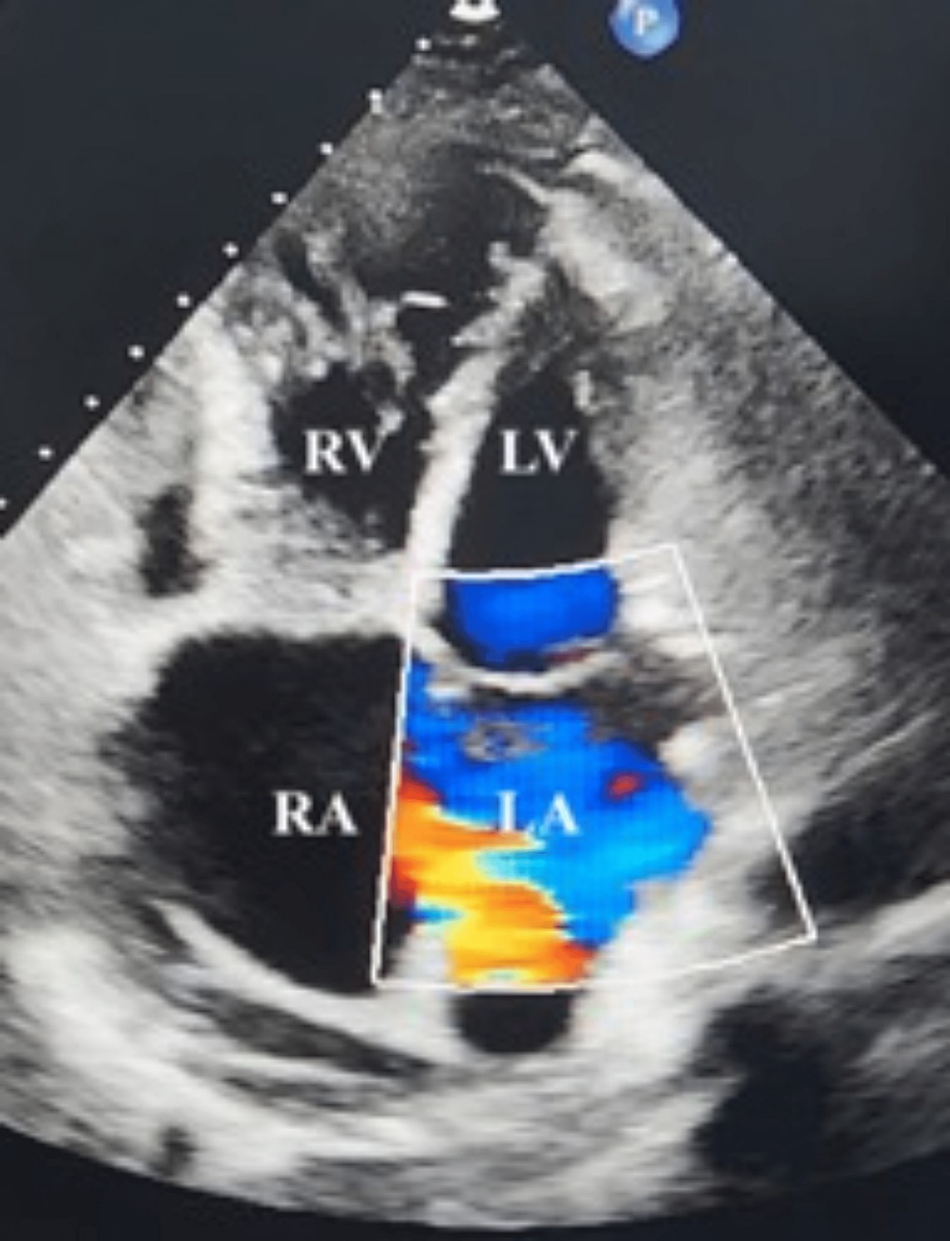
Pre-closure echocardiogram This is a four-chamber view showing the atrial septal defect (ASD) with color flow jet. LA, left atrium; LV, left ventricle; RA, right atrium; RV, right ventricle.

In view of unresolving symptoms, she presented to our emergency department eight days later. She was noted to be in moderate respiratory distress. The initial physical exam was remarkable for elevated jugular venous pressure, tachycardia, and distant heart sounds. Vitals signs were as follows: temperature 97.2 degrees Fahrenheit, heart rate 132 beats per minute, blood pressure 119/78 mmHg, oxygen saturation 98% on room air, and respiratory rate 16. Pulsus paradoxus was positive (>20 mmHg drop in systolic blood pressure with inspiration). Auscultation of the lungs revealed diffuse bilateral crackles and lower extremities with trace edema. Chest X-ray demonstrated an enlarged cardiac silhouette and vascular distension with basilar interstitial opacities. Polymerase chain reaction (PCR) testing for COVID-19 was negative. Electrocardiogram (ECG) showed sinus tachycardia with nonspecific ST-T-wave changes and significantly low amplitude with the right bundle branch block (Figure 1). Bedside echocardiogram in the emergency department was remarkable for pericardial effusion with echocardiographic and Doppler findings concerning for tamponade physiology (Figure 2).

Cardiology was immediately consulted; they recommended emergent pericardiocentesis. A pericardial drain was placed following the removal of 800 milliliters of bloody pericardial fluid. The patient was admitted to the intensive care unit (ICU) after the procedure. Two days later, the patient’s vital signs stabilized, and she was transferred from the ICU to a medical-surgical telemetry ward. At the time of her transfer out of the ICU, the patient stated that her shortness of breath had resolved, but her cough was still bothering her intermittently. She was in no respiratory distress breathing on room air and stated that she felt ready to return home. The patient reported minimal pain at the site of her pericardial drain. She denied any other symptoms at that time.

**Figure 3:**
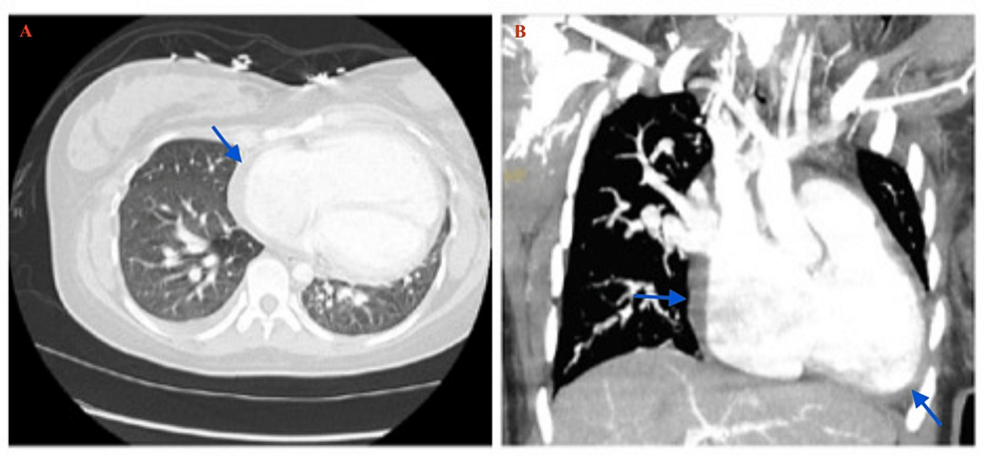
CT angiography (CTA) Chest This is a CTA chest demonstrates a trace pericardial effusion along with atrial septal defect (ASD).  Figure A is the CTA on the axial plane. Figure B is the coronal view of the CTA.  The blue arrows indicate the trace pericardial effusion. The image was done after pericardiocentesis, in preparation for ASD repair and tricuspid valve annuloplasty.

Transthoracic echocardiogram after pericardial drainage showed resolution of pericardial effusion/tamponade, a small left ventricle, a severely dilated right ventricle, severe tricuspid regurgitation, and an incidental atrial septal defect. The patient also underwent a CT angiography (CTA) of the chest (Figure 3A, 3B). Pericardial fluid studies of gram stain and culture with and without acid fast staining and fungal smear and culture were unremarkable. Serology studies for cytomegalovirus (CMV), Epstein Barr virus (EBV), human immunodeficiency virus (HIV), Influenza B, rubella, and toxoplasma were negative. Influenza type A antigen was positive. The patient was also worked up for autoimmune diseases. Rheumatoid factor, antinuclear antibody (ANA), antineutrophil cytoplasmic antibodies (C-ANCA and P-ANCA), and complement C3 and C4 were unremarkable.

As the patient’s pericardial fluid re-accumulated, a shared decision was made to undergo a surgical atrial septal defect (ASD) repair with bovine pericardium and tricuspid valve annuloplasty.

**Figure 4:**
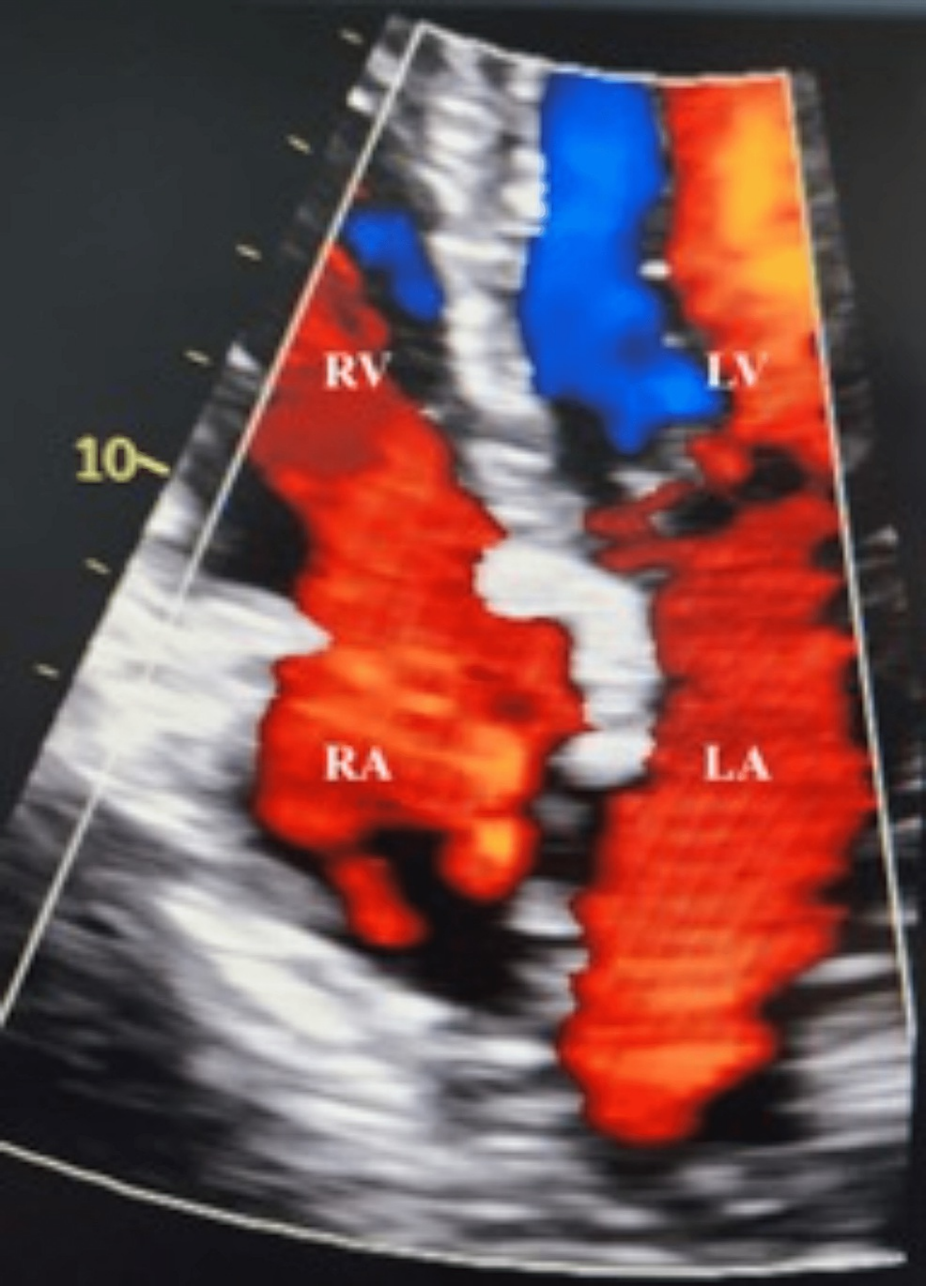
Post-closure echocardiogram This is a four-chamber view showing postoperative atrial septal defect (ASD) repair, tricuspid valve repair, and left atrial appendage ligation. As noted in the image, there is no mixing of color across the atrial septum confirming the successful closure of ASD. LA, left atrium; LV, left ventricle; RA, right atrium; RV, right ventricle.

After medical optimization, the patient underwent ASD repair with repair of the tricuspid valve and ligation of the left atrial appendage. A biopsy of the pericardium was taken during the procedure which showed benign fibro-adipose and muscular tissue with mild chronic inflammation. No malignancy was identified. The patient improved and followed up with cardiology as an outpatient. Figure 4 shows the post-closure echocardiogram.

## Discussion

This report describes the successful definitive treatment of cardiac tamponade in the setting of right ventricular failure due to a congenital ASD, possibly exacerbated by an underlying influenza type A infection.

The pericardium is a fibroelastic sac surrounding the heart, containing serous fluid with a normal volume ranging from 10 to 50 mL. A pericardial effusion is considered to have occurred when additional fluid accumulates within the sac, and hemodynamic symptoms are usually related to the rate rather than the amount of fluid accumulation. There are many etiologies that affect the pericardium in such a way that can lead to an effusion. Some of these causes are acute pericarditis due to a viral, bacterial, autoimmune/connective tissue disease (i.e. lupus), trauma, postmyocardial infarction, malignancy, mediastinal radiation, uremia in the setting of end-stage renal disease (ESRD), aortic dissection into the pericardium, idiopathic and rarely, congenital heart defects [5-7]. Although the etiology may vary in acute pericarditis, the inflammatory response, if left untreated, may lead to fluid accumulation and the development of pericardial effusion. 

A pericardial effusion may develop acutely or more insidiously. The pericardial sac can stretch and accommodate the increase in volume, dependent on the timeframe and rate of accumulation. However, with an increase in pericardial fluid volume, the intrapericardial pressure also increases and may lead to impaired cardiac function and hemodynamic compromise [2,3].

The diagnosis of cardiac tamponade is more of a clinical one, requiring prompt recognition and treatment to prevent cardiovascular collapse and cardiac arrest. The classic symptoms of cardiac tamponade were first described by the American cardiothoracic surgeon, Claude Beck, in 1935. These symptoms, known as Beck’s Triad, are hypotension, decreased or muffled heart sounds, and jugular venous distention. Although this symptomatology is highly suggestive of the presence of tamponade physiology, only a small number of cases will have all three elements [3,4]. In the case presented in this paper, the patient had muffled heart sounds, jugular vein distention, and mild peripheral edema on admission. Other signs and symptoms may also be present in such patients such as dyspnea, syncope, or pulsus paradoxus [5]. 

Once there is clinical suspicion for cardiac tamponade from physical examination, further investigation with ECG and chest radiograph may suggest the presence of pericardial effusion. However, additional imaging is usually required. Echocardiography confirms the diagnosis, as established by the 2015 European Society of Cardiology guidelines for the diagnosis and management of pericardial disease [7].

As in our case, the size of the pericardial effusion can cause hemodynamic consequences, requiring treatment by urgent pericardiocentesis. In doing so, the intrapericardial pressure is diminished, ideally restoring hemodynamic stability and decreasing the risk of cardiac arrest. Pericardiocentesis and surgical drainage of effusion are highly effective at the removal of fluid with associated hemodynamic instability. When using an indwelling catheter, it is usually let in place until fluid output is less than 25 mL per day [7]. Supportive care with fluid resuscitation with or without inotropic support may prove to be temporarily beneficial but should not be considered a substitute to pericardiocentesis. Analysis of the pericardial fluid should be taken into consideration if no obvious cause of the effusion is initially noted. As reported in our case, our patient had two underlying risk factors for tamponade physiology: a congenital ASD and a positive influenza type A antigen.

As previously mentioned, congenital atrial septal defects increase the risk of developing a pericardial effusion. In a retrospective study, analyzing the prevalence of pericardial effusions in children with ASD compared to those with ventricular septal defect (VSD) and those with normal cardiac anatomy, 32% of children (n=81) with a known ASD also had an effusion present in the pericardial space [8]. In addition, it is known that infections of the myocardium may induce an inflammatory response, leading to the accumulation of fluid in the pericardium. It is difficult to discern if our patient developed her large pericardial effusion as a complication of her recent influenza A infection or whether it was related to her ASD and right-sided heart failure. It may be likely that she had an underlying pericardial effusion which was acutely exacerbated by the viral illness, leading to the worsening of her symptoms and ultimate presentation to a healthcare facility.

The ASD in our patient was successfully repaired, along with the repair of the tricuspid valve and ligation of the left atrial appendage. It is worth noting that there have been reported paradoxical cases of tamponade after ASD closure [9,10]. In a prospective assessment, it was noted that pericardial effusions occurred commonly after open heart surgery for congenital repairs. The average onset of effusions was 7 days post-surgery. Post-surgical monitoring up to 28 days postoperatively is indicated in high-risk patients. Our patient was closely monitored after surgery and was reported to be doing well during her follow-up visits in the outpatient cardiology setting.

A congenital ASD is a chronic condition that can go asymptomatic and undiagnosed for years. It is a rare cause of pericardial effusion and tamponade, due to the chronicity of this disease, and the slow rate of accumulation of fluid. A viral illness, on the other hand, is more acute and can lead to pericardial effusion, and possibly tamponade much faster, due to the increased rate of fluid accumulation.

Our case is a good illustration of the possible multiple etiologies for pericardial effusion, as well as the pathophysiology of tamponade. We would like to emphasize the importance of using the appropriate diagnostic tools such as EKG, chest X-ray, echocardiogram, CT, and laboratory studies to find the underlying etiology, as in our case, without neglecting the basic clinical exam skills.

## Conclusions

We present an interesting case of a young female patient who presented with a large pericardial effusion and tamponade physiology unmasking a large ASD causing right ventricular failure. It is difficult to discern whether the patient developed the large pericardial effusion as a complication of her recent influenza A infection or whether it was related to her ASD and right-sided heart failure versus both. This report offers insight into the management of a patient who presents with both conditions and how this could be difficult to diagnose given the absence of the classic findings of cardiac tamponade in patients with ASD.
